# Distribution and Abundance of the Tick-Borne Piroplasm *Theileria cervi* Parasitizing White-Tailed Deer (*Odocoileus virginianus*) in Massachusetts

**DOI:** 10.3390/pathogens15080869

**Published:** 2026-08-20

**Authors:** Jacqueline O. Borges, Patrick Pearson, Guang Xu, Nolan Stamborski, Patrick Roden-Reynolds, Martin J. R. Feehan, Stephen M. Rich

**Affiliations:** 1 Laboratory of Medical Zoology, Department of Microbiology, University of Massachusetts, Amherst, MA 01002, USA; jborges@umass.edu (J.O.B.);; 2 Martha’s Vineyard Tick-Borne Illness Reduction Initiative, Edgartown, MA 02539, USA; 3 Massachusetts Division of Fisheries and Wildlife, Westborough, MA 01581, USA; martin.feehan@mass.gov; 4 Department of Natural Resources and the Environment, Cornell University, Ithaca, NY 14853, USA

**Keywords:** tick-borne diseases, pathogen surveillance, white-tailed deer, protozoan parasites, PCR, Oxford nanopore sequencing

## Abstract

Pathogen surveillance contributes to the prediction of outbreaks and implementation of strategies to minimize disease transmission. White-tailed deer (*Odocoileus virginianus*) host important disease vectors and often support the proliferation of associated pathogens. Such vectors include ticks: hematophagous acarids that facilitate pathogen transmission between animals through blood feeding. Piroplasmida, an order of blood-borne protozoa including *Babesia* and *Theileria*, exploits this feeding to infect definitive hosts. Accordingly, we investigated the prevalence of *Theileria cervi* infections in Massachusetts white-tailed deer. Conventional polymerase chain reaction (PCR) was used to detect parasite gene fragments encoding the small ribosomal subunit (18S rRNA). Confirmation of species identity was determined by Oxford nanopore sequencing and Sequencher analysis. This revealed a 32.6% prevalence of *T. cervi* across Massachusetts. Whilst surveillance of deer blood uncovers parasite abundance and distribution, inversely, this allows us to also monitor vector populations. As *Amblyomma americanum* is the only vector implicated in the transmission of *T. cervi*, the detection of *T. cervi* in regions without anticipated *Amblyomma* presence may indicate expansion of vector populations. These findings provide valuable insights into the prevalence and ecology of this piroplasm and its vector, with possible implications for veterinary and public health.

## 1. Introduction

In the United States, incidence of tick-borne diseases is on the rise [1]. In New England alone, there are seventeen known species of hard-bodied tick, several of which have been documented to parasitize humans [2]. The ability of ticks to feed on multiple vertebrates in their lifetime permits the transfer of pathogens from one individual to another. Many ixodid tick species rely on cervids as reproductive hosts. As such, cervid populations promote the proliferation of ticks, which may harbor and transmit a variety of microbes to both wildlife and humans [3]. Cervids are the most prominent ruminants in the Americas, with white-tailed deer (*Odocoileus virginianus*) being the most prevalent cervid species in the United States. White-tailed deer are greatly influenced by human activity. Increasing human populations, land development, and habitat encroachment have increased contact between humans and deer [4]. With white-tailed deer populations steadily expanding in quantity and distribution, there is a broadening opportunity for shared ectoparasite burden and resulting transmission of pathogens. As such, the development and application of pathogen and vector surveillance tools is vital.

White-tailed deer are reservoirs for a variety of understudied microbes, some of which are pathogenic to other cervids, wild and domesticated animals, and potentially humans. One such order of microbes is Piroplasmida, a group of parasitic protozoa that infects a broad range of mammals. A member of this order which infects white-tailed deer is *Theileria cervi*, which also infects several other members of the cervid family [5,6,7,8]. Upon the introduction of sporozoites to a host, the protozoa disseminate through the circulatory system. Unlike related *Babesia* species, *T. cervi* infects and lyses both leukocytes and erythrocytes, causing a disease known as theileriosis [5,6]. Infected individuals may experience anemia, lethargy, and jaundice, derived from parasitic lysis of blood cells. Most infections in white-tailed deer are subclinical, though infections in naïve individuals as well as infections accompanied by co-morbidities and environmental stressors can be fatal [9,10,11,12].

**Figure 1:**
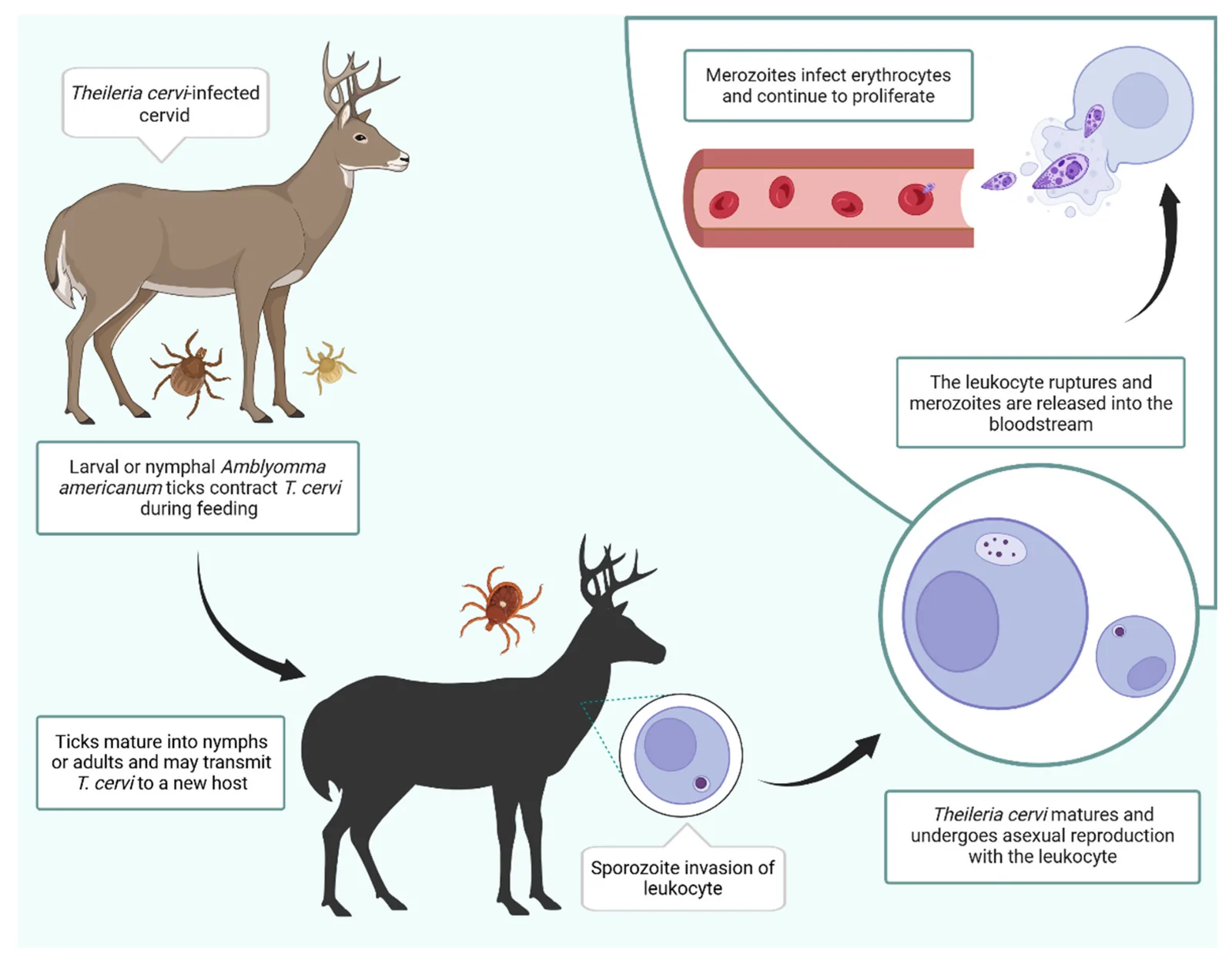
Diagram of the life cycle of *Theileria cervi.* The parasite *T. cervi* is transmitted by the tick *A. americanum* during feeding [13]. During the first stage of infection, sporozoites infect leukocytes, where they mature and replicate via merogony. The leukocytes then rupture and release merozoites. Leukocyte transformation is not known to occur during infection with *T. cervi*. Merozoites infect erythrocytes and continue to proliferate throughout the bloodstream [6,20]. Created in BioRender. Borges, J.O. (2026) https://BioRender.com/np287io.

Several parasite species propagate in and are transmitted by ixodid ticks, including the lone star tick, *Amblyomma americanum*, which introduce pathogens into the bloodstream during feeding (Figure 1). Notably, *A. americanum* are competent vectors of significant human pathogens including *Ehrlichia* spp., *Francisella*, Heartland virus, and Bourbon virus, which can cause severe illness requiring hospitalization [1]. The protozoan parasite *T. cervi* is transmitted by *A. americanum* [13]. The range of *A. americanum* has historically been restricted to the southern United States following deforestation and the loss of hosts in the northern regions of its distribution [14,15]. As a result of range expansion, *A. americanum* is now found in coastal regions of New England and is predicted to encroach further into the United States and Canada [14,16]. In Massachusetts, *A. americanum* can be found in coastal areas such as Cape Cod and the islands of Martha’s Vineyard and Nantucket [14,17]. As a singular vector for *T. cervi,* the surveillance of this piroplasm may inform predictions of the expansion of *A. americanum* and its multitude of corresponding pathogens.

This study seeks to determine the prevalence of the piroplasm *T. cervi* in Massachusetts white-tailed deer populations. This will improve our understanding of enzootic cervid disease and reveal geographic dispersal of *A. americanum* tick populations. Importantly, this research may help to validate a method for tracking the distribution of *A. americanum* in New England. Blood can be conveniently sampled from hunter-harvested deer and assessed via diagnostic tests for the presence of *T. cervi.* Positive *T. cervi* test results suggest the presence of the vector within the vicinity of where the white-tailed deer blood sample originated. The ability to assess distribution is relevant, as *A. americanum* can transmit notable human pathogens. Additionally, piroplasms cause chronic, clinical infections that impact cervid, livestock, companion animal, and human health, all of which have their own implications for the ecosystem, agriculture, and medicine. Infection with *T. cervi* is known to cause clinical symptoms in young and compromised white-tailed deer, as well as disease in other cervids [6,10,11,12]. Surveillance of *T. cervi* infections may also reveal prevalence of *Babesia odocoilei,* a related piroplasm which is significant due to its ability to cause disease in several animals and its recent detection in clinically presenting human patients [18,19]. As such, the presence of these parasites in white-tailed deer may indicate risk for local individuals. This research is especially important in New England, where humans and other animals are at a close interface with white-tailed deer and their associated ectoparasites.

## 2. Materials and Methods

### 2.1. Collection of Blood and Ticks

Blood samples were collected from hunter-harvested white-tailed deer during the 2024 hunting season. For all deer sampled, the following data was collected: date, time, location, Mass Wildlife confirmation/seal number, and the number of samples from each deer. The harvest sites were recorded for each sample (Table S1). Sampling locations were distributed throughout inland Massachusetts, where *A. americanum* are not established, and the island of Martha’s Vineyard, where *A. americanum* are established. We utilized existing MassWildlife check stations, coolers, and venison processors to conduct the sampling as a collaborative effort between hunters, processors, MassWildlife staff, volunteers, and members of the Laboratory of Medical Zoology. White-tailed deer blood was collected from nine sampling sites in inland Massachusetts: Montague, Palmer, Belchertown 1, Belchertown 2, Webster, Ayer, Hopkinton, Middleton, and Middleborough. Blood was also collected from three sampling sites on Martha’s Vineyard: West Tisbury 1, West Tisbury 2, and Manuel F. Correllus State Forest.

Blood was collected by pressing a clean Whatman filter paper (Cytiva, Marlborough, MA, USA) into the wounds and body cavity of the white-tailed deer. Saturated filter papers were stored in quart Ziploc bags (SC Johnson Professional USA, Inc., Charlotte, NC, USA), each with a unique identifier, which were left partially open for ventilation before samples could be dried. To dry the samples, filter papers rested overnight on fresh paper towels at room temperature. After drying, samples were stored at −80 °C with desiccation packets until further processed. Prior to extraction, dried blood spots (DBS) were procured from the blood-saturated filters using a sterile hole puncher, which created 6.0 mm diameter tissue chads containing approximately 25 µL of blood. For each sample, one DBS was added to a 2.0 mL SafeLock tube (Eppendorf, Hamburg, Germany) and stored again at −80 °C.

Using forceps, ticks were collected from each harvested deer for at least two minutes targeting the head and neck. Ticks were stored in ventilated vials, which were prepared by punching a hole into the lid of a plastic 7-dram vial (Fisher Scientific, Waltham, MA, USA) and adding a mesh insert to prevent tick egress through the opening. Ticks were kept in separate vials corresponding to the deer from which they were sampled. Vials were stored in a plastic bag with a damp paper towel at 4 °C until they could be further processed. Once brought to the lab, ticks were morphologically identified and the following information was recorded: genus and species, life stage, and sex. The dichotomous keys by Yunker et al. [21], Cooley and Kohls [22], and Keirans and Clifford [23] were used for tick identification. Due to the large volume of collected *Ixodes* ticks, these specimens were identified to the genus level via the position of the anal groove [23]. Locally, the species *I. scapularis* parasitizes white-tailed deer, while other *Ixodes* species have different preferred hosts [2].

### 2.2. Nucleic Acid Extraction of Dried Blood Spots

Nucleic acids were extracted from each DBS. A “blank” 6.0 mm punch from a sterile filter paper was extracted with each sample set as a negative control to monitor for cross-contamination during the punching process. Extractions were performed in sets of 30, which consisted of 28 individual blood spot samples, a blank, and an empty tube to serve as a negative control for the nucleic acid extraction.

Erythrocyte waste was removed from samples via red cell lysis. A volume of 150 µL Red Cell Lysis solution (LGC Biosearch Technologies, Hoddesdon, UK) was added to each blood spot, blank, and extraction control tube. Tubes were then thoroughly vortexed and incubated at room temperature for 5 min. Samples were then vortexed and incubated again. Leukocytes were pelleted through centrifugation at 14,000 rpm for 25 s and the supernatant containing erythrocyte was discarded.

Remaining cellular waste was removed from the samples via tissue and cell lysis. Each sample and control was treated with 1 µL of Proteinase K (LGC Biosearch Technologies) and 300 µL Tissue and Cell Lysis Solution (LGC Biosearch Technologies). Tubes were incubated at 65 °C for 15 min with vortexing every 5 min and were then cooled at 4 °C for 10 min. A volume of 150 µL MPC Precipitation Reagent (LGC Biosearch Technologies) was added to each sample and vortexed thoroughly. Cellular waste was pelletized by centrifugation for 20 min at 14,000 rpm. Soluble nucleic acids were transferred to a tube containing 500 µL absolute isopropanol, which was then inverted to wash the extracted nucleic acids. The solution was centrifuged to pellet the genetic material. Pellets were rinsed with 1000 µL 75% ethanol and dried in a 65 °C incubation oven for 20 min. The dried pellets were then resuspended in 60 µL molecular-grade water and stored at −20 °C until their use as template in polymerase chain reaction (PCR).

### 2.3. Conventional Polymerase Chain Reaction

PCR was used to amplify target host and pathogen DNA for downstream analysis. Each batch of PCRs included a no-template control for every 28 samples, which utilized molecular-grade water in place of the DNA template. Amplification was performed in 25 µL reactions. PCR master mix was prepared by combining 12.5 µL Promega G2 Colorless Master Mix (Promega, Madison, WI, USA), 9.5 µL molecular-grade water, 0.5 µL forward primer (10 µM), 0.5 µL reverse primer (10 µM), and 2 µL template DNA. Primers were ordered via Integrated DNA Technologies, Inc. (Coralville, IA, USA). Samples were amplified using Eppendorf Mastercycler pro S and epgradient S thermal cycler machines (Eppendorf).

Amplification products were separated by gel electrophoresis on a 1% agarose gel prepared with 0.5× Tris/Borate/EDTA (TBE) buffer and processed at 150 V for 45 min. Amplicon bands were visualized under both UV302 and blue light using an Axygen Gel Documentation System (Axygen, Inc., Union City, CA, USA).

To account for the quality of sample preparation, extraction, and PCR, amplification of host DNA was conducted using the primers ACTB_WTD_Ex3_F and ACTB_WTD_Ex3_R, which were designed using a shotgun sequence of *O. virginianus* chromosome 33 from GenBank (accession number: NC069706) (Table 1). These primers targeted the *O. virginianus* β-actin gene and generated an amplicon 436 bp in length. Nucleic acid extracts underwent an initial denaturation step for 2 min at 95 °C. Then, samples underwent 35 cycles of 30 s denaturation at 95 °C, 30 s annealing at 55 °C, and 40 s extension at 72 °C (Table 1). The primer annealing temperature was optimized for host PCR via gradient PCR. Three known host-positive samples were amplified using 12 different annealing temperatures that ranged from 50 to 60 °C. Once processed by gel electrophoresis, it was revealed that the amplification produced the highest-resolution bands at 55 °C, which was the annealing temperature used for the remainder of amplification.

Only blood extracts with amplified host DNA were screened for pathogen DNA. Amplification of *T. cervi* DNA was conducted using primers and parameters designed by Casati et al. [24]. The primers BJ1 and BN2 were originally utilized to amplify a fragment encoding the V4 hypervariable region of the 18S rRNA gene in *B. microti*, *B. divergens*, and *Babesia* sp. EU1 ranging from 411 to 452 bp [24]. However, these primers also anneal to the 18S rRNA gene in *T. cervi* and *B. odocoilei*, as noted by Berkley [25] and Scott et al. [19]. Amplification of this gene in *T. cervi* generates a fragment approximately 500 bp in length. Initial denaturation spanned 5 min at 94 °C. This was followed by 35 cycles of 94 °C denaturation for 1 min, 55 °C annealing for 1 min, and 72 °C extension for 2 min. Amplification concluded with a final extension at 72 °C for 5 min (Table 1).

### 2.4. Oxford Nanopore Sequencing

Due to the potential amplification of 18S fragments from multiple species, Oxford nanopore sequencing via Plasmidsaurus (South San Francisco, CA, USA) was utilized to generate a consensus sequence of the highest copy fragment in each sample. A total of 139 amplified samples were sent to Plasmidsaurus for the linear PCR sequencing service. Of these samples, 44 were from inland Massachusetts and 95 were from Martha’s Vineyard. PCR amplicons were processed with Exo-SAP-IT reagent according to manufacturer’s instructions (Thermo Fisher Scientific) prior to sequencing. Returned sequences were processed using Sequencher DNA Sequence Analysis software version 5.4.6 (Gene Codes Corporation, Ann Arbor, MI, USA), and bases with low confidence scores were trimmed from the ends of the sequences. Corresponding 18S sequences were imported from GenBank (National Institutes of Health, Bethesda, MD, USA) and assembled into contigs with sequences from Plasmidsaurus. Assembly parameters for contig formation required a minimum of 95% base match between sequences. The reference sequences included 18S fragments from *T. cervi*, *B. odocoilei*, *B. divergens*, and *B. microti* (respective GenBank accession numbers: AY735127, AY046577, AJ439713, and AF231348). When sequences did not align in Sequencher, they were identified via NCBI nucleotide BLAST version 2.17.0+ (National Institutes of Health), and the most similar sequence was imported into Sequencher for confirmation. Identified sequences were submitted to GenBank. Accession numbers for each sample can be found in Supplementary Materials (Table S1).

## 3. Results

### 3.1. Collection of Blood and Ticks

During the 2024 deer hunting season, sampling efforts yielded 297 blood spots collected from twelve sampling sites. Of these, 201 samples were derived from sites in inland Massachusetts and 96 were derived from coastal Massachusetts sites. The total collected blood samples from each site are as follows: Montague (*n* = 18), Palmer (*n* = 45), Belchertown 1 (*n* = 11), Belchertown 2 (*n* = 21), Webster (*n* = 18), Ayer (*n* = 27), Hopkinton (*n* = 29), Middleton (*n* = 8), and Middleborough (*n* = 24), West Tisbury 1 (*n* = 45), West Tisbury 2 (*n* = 12), and Manuel F. Correllus State Forest (*n* = 39).

In addition to 297 blood samples, a total of 1883 ticks were collected from the harvested deer. Morphological identification using dichotomous keys revealed the ticks to be *Ixodes* sp. (*n* = 1738), *Dermacentor albipictus* (*n* = 115), *Dermacentor* sp. (*n* = 1), and *A. americanum* (*n* = 29). One tick, denoted “*Dermacentor* sp.,” could not be identified to species level, as the distinguishing features were damaged. Importantly, *A. americanum* were only collected from sampling sites on Martha’s Vineyard, being West Tisbury 1 (*n* = 21), West Tisbury 2 (*n* = 1), and Manuel F. Correllus State Forest (*n* = 7).

### 3.2. Host DNA Amplification for PCR Internal Control

After the initial PCR targeting host DNA, the majority of the DBS yielded amplified products. As some samples did not amplify, inhibition of PCR due to the presence of heme was suspected. Negative samples underwent a series of treatments in an effort to produce host-derived amplicons. The samples were re-punched and re-extracted with double the volume of Red Cell Lysis solution. Samples that were still negative were then diluted to half of their original concentration using molecular-grade water. This restored amplification in the majority of the remaining samples. Host DNA was amplified from 98% of the DBS samples. Re-extracted and diluted samples were then used for *T. cervi* 18S PCR. The six samples that failed to amplify were not used in further experiments.

### 3.3. Parasite 18S PCR

A total of 291 DBS extracts underwent PCR for detection of *T. cervi*. Of the total samples screened, 139 samples produced bands of approximately 500 bp (47.8%). This comprised 95 positives from Martha’s Vineyard (32.6%) and 44 positives from inland Massachusetts (15.1%). Of the 96 samples from Martha’s Vineyard, 98.9% were positive. Of the 195 samples from inland Massachusetts, 22.6% were positive. Positive samples were reserved for sequencing to confirm identity.

### 3.4. Oxford Nanopore Sequencing of PCR Amplicons

**Figure 2:**
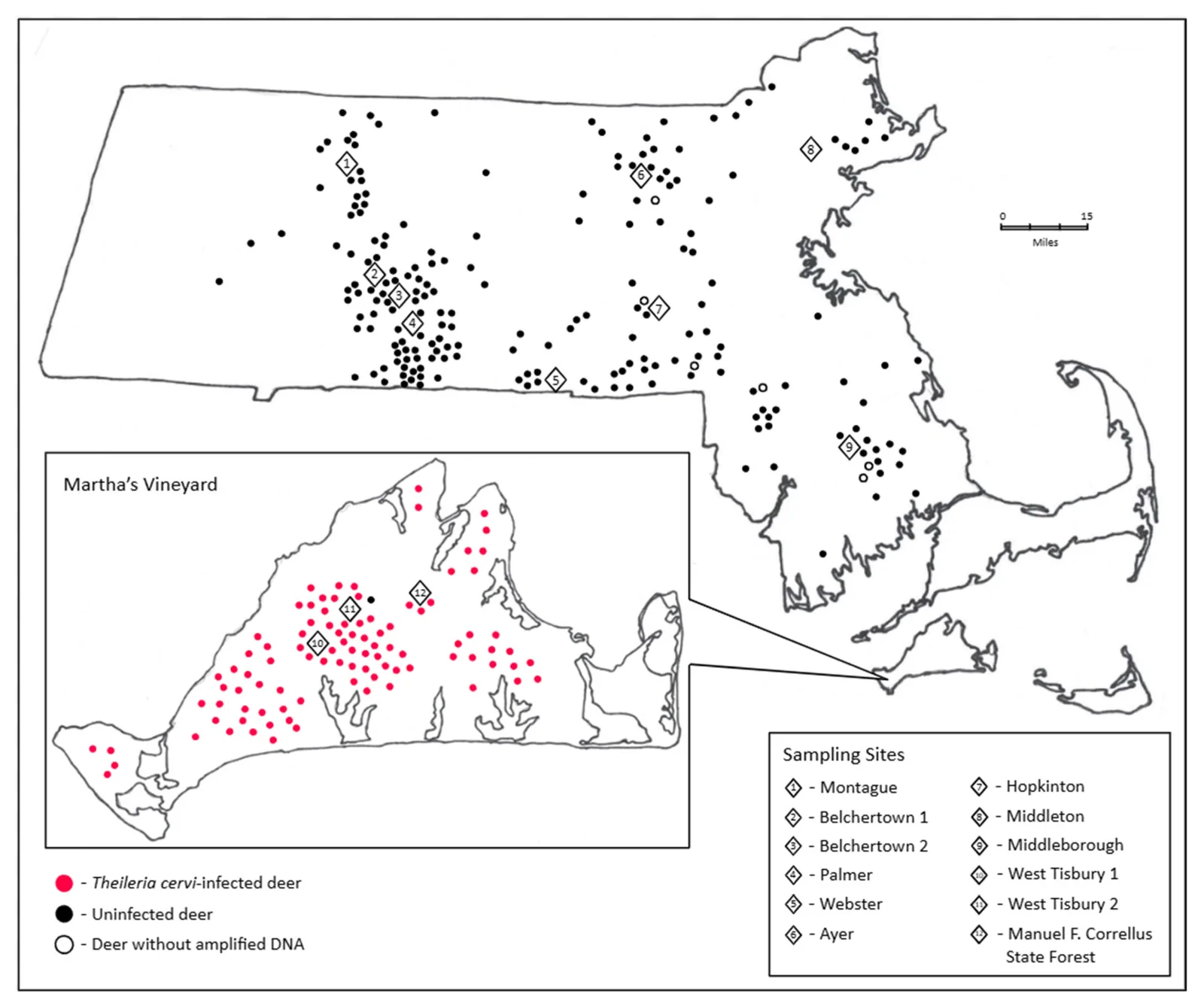
Blood was collected from deer at twelve sites across Massachusetts, including nine from inland Massachusetts and three from Martha’s Vineyard. Samples were sourced from MassWildlife locations, local hunting coolers, and venison processors. Numbered diamonds indicate sampling sites. Red dots indicate *T. cervi*-positive deer. Black dots indicate uninfected harvested deer. White dots indicate harvested deer without internal control or piroplasm PCR data. Dots are placed where deer were harvested by hunters (Table S1). Some samples lack exact hunter harvest location data.

A total of 139 amplified samples were sent to Plasmidsaurus for Oxford nanopore sequencing. Analysis of the returned sequences with Sequencher DNA Sequence Analysis software along with NCBI nucleotide BLAST, revealed that 95 samples matched documented *T. cervi* 18S gene fragments. These samples were all collected from Martha’s Vineyard, with no detection of *T. cervi* in samples from the inland sites. Thus, in coastal regions with established *A. americanum* infestations, there was a *T. cervi* prevalence of 98.9%. Of the 291 surveyed deer, 32.6% were infected with *T. cervi* (Table 2 and Figure 2).

Analysis of the remaining 44 sequences, all from inland Massachusetts samples, revealed off-target amplification. Of these, 33 sequences were identified as *B. odocoilei*, corresponding to a prevalence of 11.3% amongst the 291 surveyed deer. Three off-target sequences were identified as *Sarcocystis* sp. (1%). *Sarcocystis* is a genus of apicomplexan parasites introduced to cervids via consumption of infected fecal matter from a definitive host [26]. The last of the sequenced samples (*n* = 8) remained unidentified or were identified as cervid 18S.

## 4. Discussion

### 4.1. Abundance of T. cervi Infections in White-Tailed Deer

Across Massachusetts, 32.6% of surveyed deer samples had amplifiable *T. cervi* DNA. This percentage rises starkly when accounting for the predicted range of the vector, *A. americanum*. Existing data suggests that the range of *A. americanum* is limited to coastal regions of New England, including Nantucket and Martha’s Vineyard [14,17]. When surveying deer populations that are within the confines of the geographic range of *A. americanum* (*n* = 96), the conservative estimate for the prevalence of *T. cervi* infections in white-tailed deer is 98.9% (95 of 96). This indicates that the majority of deer on Martha’s Vineyard have *T. cervi* infections. Importantly, we only detected *T. cervi* in samples from Martha’s Vineyard, while samples from inland Massachusetts were positive for other apicomplexan parasites. Our results mirror the findings of previous research, which indicate high *T. cervi* infection rates in regions with *A. americanum* [25,27,28]. Berkley [25] found that 22 of 412 (5.3%) samples collected from deer in New England were positive for *T. cervi.* It was noted that 49 samples were collected from areas with predicted *A. americanum* inhabitance. All 22 positives were sourced from these locations, establishing a *T. cervi* prevalence of 44.9% in regions with *A. americanum* [25]. Notably, we found a significantly higher prevalence of *T. cervi* in *Amblyomma*-endemic locations. Discrepancies between the discovered infection rates may be attributed to differences in sampling size and location, or variations in deer and vector density at sampling locations. Results similar to ours have been found across the southern United States: Thompson et al. [27] reported that 189 of 293 deer (64.5%) were *Theileria*-positive while another 47 deer (16%) were *Babesia*-positive. Deer blood samples were sourced from living and hunter-harvested white-tailed deer primarily from Virginia, with some samples being from eleven other southern states. These samples were screened for piroplasm infection using 18S rRNA PCR and restriction fragment length polymorphism (RFLP) assays [27]. In a Florida surveillance study performed by Cauvin et al. [28], which used a separate 18S rRNA PCR assay, researchers found that 97.6% of sampled wild white-tailed deer were infected with *T. cervi*. This study also utilized blood collected from both living and hunter-harvested wild deer, as well as blood collected from farmed white-tailed deer. The farmed animals had a lower rate of *T. cervi* infection at 40.4% [28].

Additionally, *B. odocoilei* DNA was amplified from a significant number of deer samples. Of the 291 tested samples, 33 yielded amplified *B. odocoilei* DNA. This comprises 11.3% of the total surveyed white-tailed deer in Massachusetts, which is a marked increase from the previously recorded prevalence of 1.9% in New England states [25]. This may be attributed to differences in sampling, extraction methods, and sequencing, as the same PCR primers and parameters were used. Interestingly, no *B. odocoilei* was detected in deer on Martha’s Vineyard, despite the tick vector *I. scapularis* being widely disseminated throughout the eastern United States. This may be a consequence of lower parasitemia in cases of babesiosis, which may result in *B. odocoilei* amplicons being outcompeted by *T. cervi* amplicons from the same sample during PCR or nanopore sequencing. As sequences are returned as a consensus of the most prominent amplicon within a sample, the sequence data for amplicons in low concentrations are not readily accessible. As such, the true prevalence of *B. odocoilei* infections in deer on Martha’s Vineyard may not be reflected by the results. Higher resolution may yet be achieved through a more refined detection assay. Utilizing a PCR assay with improved specificity, such as with *Babesia*- or *Theileria*-specific primers, could significantly enhance detection and downstream genetic analysis. Cloning could isolate different amplicons within a sample but cannot be reasonably applied to large sample sets with indeterminate proportions of amplicons.

### 4.2. Geographic Distribution of Amblyomma americanum

We found that *T. cervi* infections are more prolific in regions which are inhabited by the vector *A. americanum*, which coincides with the findings of Berkley [25]. This was further supported by the tick collection data from this study. All *A. americanum* were collected from sites on Martha’s Vineyard, while other tick species were collected broadly across Massachusetts. This correlation suggests that detection of *T. cervi* in harvested white-tailed deer blood may be used as a proxy sentinel to record the expansion of *A. americanum*. There were relatively few *A. americanum* specimens collected for the number of white-tailed deer surveyed on Martha’s Vineyard, which may be attributed to the time period during which sampling occurred. In the northeastern U.S., nymphal and adult *A. americanum* activity is documented to peak between May and July [17], with larval activity extending into September [29]. During these months, greater densities of ticks may be observed on white-tailed deer, which has been documented in other regions [30]. Hunting season spans from October to December, thus our screening of hunter-harvested white-tailed deer does not capture the greatest *A. americanum* burdens for optimal direct surveillance. However, this approach does ensure that deer have experienced at least one season of peak *A. americanum* activity. While unfed tick sampling is well-described and offers direct detection, difficulties in locating sparse *A. americanum* at the frontiers of expansion may limit the practicality of this endeavor. Additionally, there are biological and physical risks posed to individuals performing field research. Applying the described molecular detection methods to hunter-harvested white-tailed deer provides a surveillance technique that is convenient and reduces exposure to hazards associated with field research.

The present sampling strategy was aimed at testing the presence or absence of *T. cervi* in distinct locations where *A. americanum* are known to be endemic or absent. Future sampling from more locations across New England would help to identify the current range of *A. americanum* and continue to monitor its expansion. It is important to note that the samples representing regions in which *A. americanum* are endemic are sourced exclusively from Martha’s Vineyard. Martha’s Vineyard, along with other Massachusetts islands, has a notably high white-tailed deer density of above 24 deer/km^2^ in areas open to hunting and far higher in areas closed to hunting [31]. This may contribute to the nearly ubiquitous (98.9%) prevalence of *T. cervi* recorded in this study.

### 4.3. The Use of Host Internal Controls for Quality Assurance

Here we used a white-tailed deer PCR internal control to improve quality of the results and reduce risk of false negative findings. This layer of PCR was initially proposed as a screening tool for all incoming DBS samples to ensure that the calculated percentage of piroplasm infections was accurately based around the number of samples with quality blood. Samples without amplified white-tailed deer β-actin DNA were presumed to have been harvested, stored, or transported in such a way that it degraded the sample. However, three samples which lacked amplification in white-tailed deer β-actin PCR, ultimately produced piroplasm 18S amplicons using BJ1 and BN2 primers. A dilution assay was conducted by diluting the DNA template by 50% before amplification, which ultimately restored amplification. This suggests that the host PCR assay is inhibited by the presence of heme impurities in the template, which may be exacerbated by suboptimal primer annealing efficiency. Edits may yet be made to the extraction techniques, primer design, and thermal cycling parameters to improve this assay as a sample quality assessment tool.

### 4.4. Detection Methods for Understudied Eukaryotic Microbes

A considerable number of blood samples had amplifiable piroplasm DNA. The utilized primer set was designed to broadly target the V4 hypervariable region of *Babesia* spp. but also amplifies this sequence in *T. cervi* [24,25]. The small ribosomal subunit is conserved across kingdoms but varies by species, thus is commonly used to survey novel microbes and genetic diversity within species. The V4 hypervariable region of 18S rRNA was considered an appropriate target as it contains hypervariable segments that are helpful in distinguishing parasite species and genotypes [24]. It was also a convenient target, as there are limited alternative gene sequences for *T. cervi* documented in the GenBank database. This primer set produced off-target amplicons (*n* = 44), which indicates that sequencing is necessary to verify the identity of amplified products. Initial trials with various primer sets produced significant off-target amplification (data not included). When analyzed with Sanger sequencing, the preponderance of mixed 18S fragments yielded uninterpretable sequences. With this challenge under consideration, Oxford nanopore sequencing was utilized to obtain readable sequences. This imposes the limitations of consensus sequence formation, which potentially permits dual- or multi-piroplasm infections to go unnoticed depending on the relative concentrations of 18S amplicons from each species. This may yet be resolved by targeting a gene with increased specificity. Currently, *T. cervi* and *B. odocoilei* genome sequences are not available in GenBank. Primers may be designed using documented sequences from related piroplasm species, though much development may be required to amplify fragments from their cervid-infecting counterparts. Culturing these organisms through whole blood may also allow for improved isolation and categorization of each species and would limit background amplification of off-target 18S fragments from the host or sample contaminants.

## 5. Conclusions

This research indicates a significant prevalence of *T. cervi* infections in Massachusetts white-tailed deer, with infections present in high concentration on the island of Martha’s Vineyard. These findings support the understood model of vector distribution and may allow for the detection of *T. cervi* as a sentinel for monitoring the expansion of *A. americanum* populations. Still, improvements are needed for future research on white-tailed deer and the understudied microbes they host. Optimization of nucleic acid extraction, primer design, and PCR parameters may improve such studies. Ideally, with adjusted screening methods, additional incidence and genetic diversity of piroplasms may be accounted for. Analysis of samples from additional sites will allow for extended surveillance of New England white-tailed deer and expansion of vector distributions. Improving our understanding of these infectious agents and the distribution of their vectors is an important undertaking for human and cervid wellness, especially in the face of continuous tick range expansion.

## Figures and Tables

**Table 1:** Primers and thermal cycling conditions for white-tailed deer and *T. cervi* gene targets. We designed the white-tailed deer β-actin gene primers to establish a PCR internal control. The V4 hypervariable region of the 18S rRNA gene was targeted in *T. cervi* PCR [24]. Annealing temperatures, extension durations, and number of cycles were adjusted based on primer melting temperatures and expected amplicon lengths.

Target Gene	Primer Information	Amplicon Length	Thermal Cycling Parameters
White-tailed deer β-actin	ACTB_WTD_Ex3_F: 5′-CATGTTCGAGACCTTCAACACC-3′ ACTB_WTD_Ex3_R: 5′-CAGGAAGGAAGGCTGGAAGAG-3′	436 bp	95 °C for 2 min 95 °C for 30 s (×35 cycles) 55 °C for 30 s (×35 cycles) 72 °C for 40 s (×35 cycles) 72 °C for 5 min
*T. cervi* 18S rRNA	BJ1: 5′-GTCTTGTAATTGGAATGATGG-3′ BN2: 5′-TAGTTTATGGTTAGGACTACG-3′	~500 bp	94 °C for 5 min 94 °C for 1 min (×35 cycles) 55 °C for 1 min (×35 cycles) 72 °C for 2 min (×35 cycles) 72 °C for 5 min

**Table 2:** Results of 18S rRNA V4 gene Oxford nanopore sequencing. Nanopore sequencing of gel-positive samples confirmed that a majority of amplicons matched known sequences from *T. cervi*. Comparisons were completed using BLAST and Sequencher software. Prevalence of *T. cervi* is represented individually based on sampling region.

Surveyed Region	Number of Surveyed Samples	*Theileria cervi*- Positive Samples
Inland Massachusetts	195	0 (0%)
Martha’s Vineyard, Massachusetts	96	95 (98.9%)
Total	291	95 (32.6%)

## Data Availability

All relevant data are included in the manuscript or Supplementary Materials.

## Supplementary Materials

The following supporting information can be downloaded at https://www.mdpi.com/article/10.3390/pathogens15080869/s1, Supplemental Table S1: White-tailed deer harvest and infection data. The available data associated with each deer used in the study, including GenBank accession numbers for samples with sequenced parasite DNA. Some data was not recorded for every individual.

## Abbreviations

The following abbreviations are used in this manuscript:

DBS	Dried blood spot
PCR	Polymerase chain reaction
MV	Martha’s Vineyard
RFLP	Restriction fragment length polymorphism
