# Supplementary material for: Distribution and Abundance of the Tick-Borne Piroplasm Theileria cervi Parasitizing White-Tailed Deer (Odocoileus virginianus) in Massachusetts
Source: Pathogens. 2026 Aug 20;15(8):869. doi: 10.3390/pathogens15080869 (PMC13516185; doi:10.3390/pathogens15080869)
Supplement: Supplementary file 1 [file pathogens-15-00869-s001.zip › pathogens-4506111-supplementary.pdf]

Supplemental Table S1. White-tailed deer harvest and infection data. The available data associated with each deer used in the study, including GenBank accession numbers for samples with sequenced parasite DNA. Some data was not recorded for every individual.

Check Station or Processor	Sample code	Town Harvested	Deer Sex	Deer Age	Amplifiation of white-tailed deer DNA	Amplification of parasite DNA	Parasite Identification	Genbank Accession
West Tisbury 1	MAWTD24_1	West Tisbury	Female	N/A	Positive	Positive	*Theileria cervi*	PZ495356
West Tisbury 1	MAWTD24_2	Chilmark	Female	N/A	Positive	Positive	*Theileria cervi*	PZ496755
West Tisbury 1	MAWTD24_3	Chilmark	Female	N/A	Positive	Positive	*Theileria cervi*	PZ496904
West Tisbury 1	MAWTD24_4	Chilmark	Male	N/A	Positive	Positive	*Theileria cervi*	PZ497147
West Tisbury 1	MAWTD24_5	West Tisbury	Male	N/A	Positive	Positive	*Theileria cervi*	PZ500533
West Tisbury 1	MAWTD24_6	West Tisbury	Male	N/A	Positive	Negative	N/A	N/A
West Tisbury 1	MAWTD24_7	West Tisbury	Male	N/A	Positive	Positive	*Theileria cervi*	PZ500592
West Tisbury 1	MAWTD24_8	Tisbury	Male	N/A	Positive	Positive	*Theileria cervi*	PZ501379
West Tisbury 1	MAWTD24_9	West Tisbury	Female	N/A	Positive	Positive	*Theileria cervi*	PZ501394
West Tisbury 1	MAWTD24_10	West Tisbury	Male	N/A	Positive	Positive	*Theileria cervi*	PZ501401
West Tisbury 1	MAWTD24_11	Edgartown	Male	N/A	Positive	Positive	*Theileria cervi*	PZ501408
West Tisbury 1	MAWTD24_12	Chilmark	Male	N/A	Positive	Positive	*Theileria cervi*	PZ501426
West Tisbury 1	MAWTD24_13	Chilmark	Male	N/A	Positive	Positive	*Theileria cervi*	PZ501437
West Tisbury 1	MAWTD24_14	West Tisbury	Female	N/A	Positive	Positive	*Theileria cervi*	PZ501456
West Tisbury 1	MAWTD24_15	West Tisbury	Male	N/A	Positive	Positive	*Theileria cervi*	PZ501480
West Tisbury 1	MAWTD24_16	Chilmark	Male	N/A	Positive	Positive	*Theileria cervi*	PZ506357
West Tisbury 1	MAWTD24_17	West Tisbury	Male	N/A	Positive	Positive	*Theileria cervi*	PZ501532
West Tisbury 1	MAWTD24_18	*West Tisbury at Site	Male	N/A	Positive	Positive	*Theileria cervi*	PZ501828
West Tisbury 1	MAWTD24_19	West Tisbury	Male	N/A	Positive	Positive	*Theileria cervi*	PZ501829
West Tisbury 1	MAWTD24_20	Oak Bluffs	Female	N/A	Positive	Positive	*Theileria cervi*	PZ501830
West Tisbury 1	MAWTD24_21	West Tisbury	Male	0.5	Positive	Positive	*Theileria cervi*	PZ501832
West Tisbury 1	MAWTD24_22	West Tisbury	Female	N/A	Positive	Positive	*Theileria cervi*	PZ501833
West Tisbury 1	MAWTD24_23	Oak Bluffs	Male	N/A	Positive	Positive	*Theileria cervi*	PZ501835
West Tisbury 1	MAWTD24_24	West Tisbury	Male	0.5	Positive	Positive	*Theileria cervi*	PZ501836
West Tisbury 1	MAWTD24_25	Edgartown	Female	0.5	Positive	Positive	*Theileria cervi*	PZ501851
West Tisbury 1	MAWTD24_26	Oak Bluffs	Male	2.5	Positive	Positive	*Theileria cervi*	PZ501863
West Tisbury 1	MAWTD24_27	*West Tisbury at Site	Female	N/A	Positive	Positive	*Theileria cervi*	PZ501867
West Tisbury 1	MAWTD24_28	Chilmark	Female	1.5	Positive	Positive	*Theileria cervi*	PZ501869
West Tisbury 1	MAWTD24_29	Chilmark	Female	3.5+	Positive	Positive	*Theileria cervi*	PZ501870
West Tisbury 1	MAWTD24_30	Chilmark	Male	0.5	Positive	Positive	*Theileria cervi*	PZ506358
West Tisbury 1	MAWTD24_31	Chilmark	Female	2.5	Positive	Positive	*Theileria cervi*	PZ501871
West Tisbury 1	MAWTD24_32	Chilmark	Female	0.5	Positive	Positive	*Theileria cervi*	PZ506359
West Tisbury 1	MAWTD24_33	*West Tisbury at Site	Female	N/A	Positive	Positive	*Theileria cervi*	PZ501894
West Tisbury 1	MAWTD24_34	Chilmark	Female	3.5+	Positive	Positive	*Theileria cervi*	PZ501895
West Tisbury 1	MAWTD24_35	Chilmark	Male	2.5	Positive	Positive	*Theileria cervi*	PZ502246
West Tisbury 1	MAWTD24_36	West Tisbury	Female	2.5	Positive	Positive	*Theileria cervi*	PZ502304
West Tisbury 1	MAWTD24_37	West Tisbury	Female	N/A	Positive	Positive	*Theileria cervi*	PZ502811
West Tisbury 1	MAWTD24_38	Chilmark	Male	N/A	Positive	Positive	*Theileria cervi*	PZ502813
West Tisbury 1	MAWTD24_39	Aquinnah	Female	N/A	Positive	Positive	*Theileria cervi*	PZ503146
West Tisbury 1	MAWTD24_40	West Tisbury	Male	N/A	Positive	Positive	*Theileria cervi*	PZ503147
West Tisbury 1	MAWTD24_41	West Tisbury	Male	N/A	Positive	Positive	*Theileria cervi*	PZ506360
West Tisbury 1	MAWTD24_42	West Tisbury	Male	N/A	Positive	Positive	*Theileria cervi*	PZ503158
West Tisbury 1	MAWTD24_43	Chilmark	Male	N/A	Positive	Positive	*Theileria cervi*	PZ506361
West Tisbury 1	MAWTD24_44	West Tisbury	Female	N/A	Positive	Positive	*Theileria cervi*	PZ503168
West Tisbury 1	MAWTD24_45	West Tisbury	Male	N/A	Positive	Positive	*Theileria cervi*	PZ503170
West Tisbury 2	MAWTD24_46	Chilmark	Female	N/A	Positive	Positive	*Theileria cervi*	PZ503194
West Tisbury 2	MAWTD24_47	West Tisbury	Female	N/A	Positive	Positive	*Theileria cervi*	PZ503281
West Tisbury 2	MAWTD24_48	West Tisbury	Female	N/A	Positive	Positive	*Theileria cervi*	PZ506362
West Tisbury 2	MAWTD24_49	Chilmark	Female	N/A	Positive	Positive	*Theileria cervi*	PZ600309
West Tisbury 2	MAWTD24_50	West Tisbury	Female	N/A	Positive	Positive	*Theileria cervi*	PZ600335
West Tisbury 2	MAWTD24_51	West Tisbury	Male	N/A	Positive	Positive	*Theileria cervi*	PZ600352
West Tisbury 2	MAWTD24_52	West Tisbury	Female	N/A	Positive	Positive	*Theileria cervi*	PZ600353
West Tisbury 2	MAWTD24_53	West Tisbury	Female	N/A	Positive	Positive	*Theileria cervi*	PZ600593
West Tisbury 2	MAWTD24_54	West Tisbury	Male	N/A	Positive	Positive	*Theileria cervi*	PZ600355
West Tisbury 2	MAWTD24_55	West Tisbury	Female	N/A	Positive	Positive	*Theileria cervi*	PZ600594
West Tisbury 2	MAWTD24_56	Chilmark	Male	N/A	Positive	Positive	*Theileria cervi*	PZ600401
West Tisbury 2	MAWTD24_57	Chilmark	Female	N/A	Positive	Positive	*Theileria cervi*	PZ600511
Manuel F Correllus	MAWTD24_58	West Tisbury	Female	1.5	Positive	Positive	*Theileria cervi*	PZ507142
Manuel F Correllus	MAWTD24_59	Edgartown	Female	1.5	Positive	Positive	*Theileria cervi*	PZ507285
Manuel F Correllus	MAWTD24_60	Edgartown	Male	0.5	Positive	Positive	*Theileria cervi*	PZ507384
Manuel F Correllus	MAWTD24_61	Chilmark	Female	N/A	Positive	Positive	*Theileria cervi*	PZ507607

Manuel F Correllus	MAWTD24_62	West Tisbury	Male	1.5	Positive	Positive	*Theileria cervi*	PZ507501
Manuel F Correllus	MAWTD24_63	*Manuel F Correllus State	N/A	N/A	Positive	Positive	*Theileria cervi*	PZ507535
Manuel F Correllus	MAWTD24_64	West Tisbury	Male	2.5	Positive	Positive	*Theileria cervi*	PZ509257
Manuel F Correllus	MAWTD24_65	Aquinnah	Female	1.5	Positive	Positive	*Theileria cervi*	PZ509268
Manuel F Correllus	MAWTD24_66	Aquinnah	Male	1.5	Positive	Positive	*Theileria cervi*	PZ509269
Manuel F Correllus	MAWTD24_67	Chilmark	Female	0.5	Positive	Positive	*Theileria cervi*	PZ509680
Manuel F Correllus	MAWTD24_68	Edgartown	Female	2.5	Positive	Positive	*Theileria cervi*	PZ509684
Manuel F Correllus	MAWTD24_69	Edgartown	Female	1.5	Positive	Positive	*Theileria cervi*	PZ510006
Manuel F Correllus	MAWTD24_70	West Tisbury	Male	0.5	Positive	Positive	*Theileria cervi*	PZ510013
Manuel F Correllus	MAWTD24_71	Edgartown	Male	3.5+	Positive	Positive	*Theileria cervi*	PZ511772
Manuel F Correllus	MAWTD24_72	Edgartown	Female	3.5+	Positive	Positive	*Theileria cervi*	PZ511773
Manuel F Correllus	MAWTD24_73	Chilmark	Male	3.5+	Positive	Positive	*Theileria cervi*	PZ510233
Manuel F Correllus	MAWTD24_74	Oak Bluffs	Male	2.5	Positive	Positive	*Theileria cervi*	PZ510381
Manuel F Correllus	MAWTD24_75	Oak Bluffs	Male	1.5	Positive	Positive	*Theileria cervi*	PZ510383
Manuel F Correllus	MAWTD24_76	Aquinnah	Male	2.5	Positive	Positive	*Theileria cervi*	PZ511774
Manuel F Correllus	MAWTD24_77	Chilmark	Female	3.5+	Positive	Positive	*Theileria cervi*	PZ510963
Manuel F Correllus	MAWTD24_78	Chilmark	Female	3.5+	Positive	Positive	*Theileria cervi*	PZ510968
Manuel F Correllus	MAWTD24_79	Oak Bluffs	Male	3.5+	Positive	Positive	*Theileria cervi*	PZ511061
Manuel F Correllus	MAWTD24_80	Edgartown	Male	1.5	Positive	Positive	*Theileria cervi*	PZ511064
Manuel F Correllus	MAWTD24_81	West Tisbury	Male	1.5	Positive	Positive	*Theileria cervi*	PZ511800
Manuel F Correllus	MAWTD24_82	West Tisbury	Male	2.5	Positive	Positive	*Theileria cervi*	PZ511801
Manuel F Correllus	MAWTD24_83	West Tisbury	Female	3.5+	Positive	Positive	*Theileria cervi*	PZ511202
Manuel F Correllus	MAWTD24_84	Edgartown	Male	2.5	Positive	Positive	*Theileria cervi*	PZ511802
Manuel F Correllus	MAWTD24_85	Edgartown	Female	3.5+	Positive	Positive	*Theileria cervi*	PZ511803
Manuel F Correllus	MAWTD24_86	Edgartown	Female	3.5+	Positive	Positive	*Theileria cervi*	PZ511498
Manuel F Correllus	MAWTD24_87	Edgartown	Female	3.5+	Positive	Positive	*Theileria cervi*	PZ511499
Manuel F Correllus	MAWTD24_88	West Tisbury	Female	2.5	Positive	Positive	*Theileria cervi*	PZ513125
Manuel F Correllus	MAWTD24_89	Chilmark	Male	0.5	Positive	Positive	*Theileria cervi*	PZ513170
Manuel F Correllus	MAWTD24_90	Chilmark	Male	2.5	Positive	Positive	*Theileria cervi*	PZ513400
Manuel F Correllus	MAWTD24_91	West Tisbury	Female	2.5	Positive	Positive	*Theileria cervi*	PZ518421
Manuel F Correllus	MAWTD24_92	West Tisbury	Female	0.5	Positive	Positive	*Theileria cervi*	PZ513413
Manuel F Correllus	MAWTD24_93	*Manuel F Correllus State	N/A	N/A	Positive	Positive	*Theileria cervi*	PZ513470
Manuel F Correllus	MAWTD24_94	*Manuel F Correllus State	N/A	N/A	Positive	Positive	*Theileria cervi*	PZ513483
Manuel F Correllus	MAWTD24_95	West Tisbury	Female	2.5	Positive	Positive	*Theileria cervi*	PZ513495
Manuel F Correllus	MAWTD24_96	Tisbury	Male	N/A	Positive	Positive	*Theileria cervi*	PZ513684
Palmer	MAWTD24_97	Monson	Male	3.5+	Positive	Negative	N/A	N/A
Palmer	MAWTD24_98	Hampden	Male	3.5+	Positive	Negative	N/A	N/A
Palmer	MAWTD24_99	Monson	Male	3.5+	Positive	Negative	N/A	N/A
Palmer	MAWTD24_100	Wales	Male	1.5	Positive	Positive	*Babesia odocoile*	PZ528232
Palmer	MAWTD24_101	Hampden	Female	1.5	Positive	Negative	N/A	N/A
Palmer	MAWTD24_102	Hampden	Male	3.5+	Positive	Negative	N/A	N/A
Palmer	MAWTD24_103	Monson	Female	2.5	Positive	Positive	*Babesia odocoile*	PZ528245
Palmer	MAWTD24_104	Monson	Male	2.5	Positive	Negative	N/A	N/A
Palmer	MAWTD24_105	Brimfield	Female	1.5	Positive	Negative	N/A	N/A
Palmer	MAWTD24_106	Warren	Female	3.5+	Positive	Negative	N/A	N/A
Palmer	MAWTD24_107	Monson	Female	3.5+	Positive	Negative	N/A	N/A
Palmer	MAWTD24_108	Monson	Male	1.5	Positive	Negative	N/A	N/A
Palmer	MAWTD24_109	Belchertown	Male	1.5	Positive	Negative	N/A	N/A
Palmer	MAWTD24_110	Ware	Female	3.5+	Positive	Negative	N/A	N/A
Palmer	MAWTD24_111	Monson	Female	3.5+	Positive	Negative	N/A	N/A
Palmer	MAWTD24_112	Hardwick	Male	2.5	Positive	Negative	N/A	N/A
Palmer	MAWTD24_113	Monson	Female	2.5	Positive	Negative	N/A	N/A
Palmer	MAWTD24_114	Hardwick	Male	3.5+	Positive	Negative	N/A	N/A
Palmer	MAWTD24_115	Ludlow	Male	3.5+	Positive	Negative	N/A	N/A
Palmer	MAWTD24_116	Palmer	Male	1.5	Positive	Negative	N/A	N/A
Palmer	MAWTD24_117	Ware	Male	2.5	Positive	Negative	N/A	N/A
Palmer	MAWTD24_118	Palmer	Male	1.5	Positive	Negative	N/A	N/A
Palmer	MAWTD24_119	Hardwick	Female	3.5+	Positive	Negative	N/A	N/A
Palmer	MAWTD24_120	Brimfield	Male	2.5	Positive	Negative	N/A	N/A
Palmer	MAWTD24_121	Warren	Female	0.5	Positive	Negative	N/A	N/A
Palmer	MAWTD24_122	Brimfield	Male	2.5	Positive	Negative	N/A	N/A
Palmer	MAWTD24_123	Chester	Male	3.5+	Positive	Negative	N/A	N/A
Palmer	MAWTD24_124	Ludlow	Male	2.5	Positive	Negative	N/A	N/A
Palmer	MAWTD24_125	Monson	Male	3.5+	Positive	Positive	*Babesia odocoile*	PZ528270
Palmer	MAWTD24_126	Brimfield	Female	2.5	Positive	Positive	*Babesia odocoile*	PZ528271
Palmer	MAWTD24_127	East Longmeadow	Male	3.5+	Positive	Positive	*Babesia odocoile*	PZ530592
Palmer	MAWTD24_128	New Braintree	Male	3.5+	Positive	Negative	N/A	N/A
Palmer	MAWTD24_129	Warren	Female	3.5+	Positive	Negative	N/A	N/A

Palmer	MAWTD24_130	Monson	Male	1.5	Positive	Negative	N/A	N/A
Palmer	MAWTD24_131	Monson	Female	0.5	Positive	Positive	*Babesia odocoile*	PZ528282
Palmer	MAWTD24_132	Ware	Female	2.5	Positive	Negative	N/A	N/A
Palmer	MAWTD24_133	Monson	Male	2.5	Positive	Negative	N/A	N/A
Palmer	MAWTD24_134	Monson	Female	2.5	Positive	Negative	N/A	N/A
Palmer	MAWTD24_135	Monson	Female	3.5+	Positive	Negative	N/A	N/A
Palmer	MAWTD24_136	Brimfield	Female	3.5+	Positive	Negative	N/A	N/A
Palmer	MAWTD24_137	Monson	Female	3.5+	Positive	Negative	N/A	N/A
Palmer	MAWTD24_138	Warren	Female	2.5	Positive	Negative	N/A	N/A
Palmer	MAWTD24_139	Palmer	Female	1.5	Positive	Positive	*Babesia odocoile*	PZ528294
Palmer	MAWTD24_140	Brimfield	Male	3.5+	Positive	Negative	N/A	N/A
Palmer	MAWTD24_141	Brimfield	Female	3.5+	Positive	Negative	N/A	N/A
Montague	MAWTD24_142	Royalston	Female	2.5	Positive	Negative	N/A	N/A
Montague	MAWTD24_143	Deerfield	Male	2.5	Positive	Negative	N/A	N/A
Montague	MAWTD24_144	Leverett	Male	3.5+	Positive	Negative	N/A	N/A
Montague	MAWTD24_145	Northfield	Male	1.5	Positive	Negative	N/A	N/A
Montague	MAWTD24_146	Greenfield	Female	3.5+	Positive	Negative	N/A	N/A
Montague	MAWTD24_147	Gill	Male	2.5	Positive	Positive	*Babesia odocoile*	PZ528295
Montague	MAWTD24_148	Montague	Male	3.5+	Positive	Negative	N/A	N/A
Montague	MAWTD24_149	Montague	Male	2.5	Positive	Negative	N/A	N/A
Montague	MAWTD24_150	Northfield	Male	2.5	Positive	Negative	N/A	N/A
Montague	MAWTD24_151	Greenfield	Female	3.5+	Positive	Negative	N/A	N/A
Montague	MAWTD24_152	Greenfield	Female	3.5+	Positive	Negative	N/A	N/A
Montague	MAWTD24_153	Gill	Male	2.5	Positive	Positive	*Babesia odocoile*	PZ528296
Montague	MAWTD24_154	Bernardston	Female	2.5	Positive	Negative	N/A	N/A
Montague	MAWTD24_155	Gill	Male	2.5	Positive	Negative	N/A	N/A
Montague	MAWTD24_156	Leverett	Female	2.5	Positive	Negative	N/A	N/A
Montague	MAWTD24_157	Leverett	Female	0.5	Positive	Positive	*Babesia odocoile*	PZ530595
Montague	MAWTD24_158	Leverett	Female	3.5+	Positive	Negative	N/A	N/A
Montague	MAWTD24_159	Montague	Male	1.5	Positive	Negative	N/A	N/A
Belchertown 1	MAWTD24_160	Hadley	Male	1.5	Positive	Negative	N/A	N/A
Belchertown 1	MAWTD24_161	North Brookfield	Male	3.5+	Positive	Negative	N/A	N/A
Belchertown 1	MAWTD24_162	Monson	Female	3.5+	Positive	Negative	N/A	N/A
Belchertown 1	MAWTD24_163	Chesterfield	Female	3.5+	Positive	Positive	*Sarcocystis* sp.	PZ623930
Belchertown 1	MAWTD24_164	*Belchertown at Site	Male	N/A	Positive	Negative	N/A	N/A
Belchertown 1	MAWTD24_165	Granby	Male	2.5	Positive	Positive	*Babesia odocoile*	PZ530594
Belchertown 1	MAWTD24_166	Belchertown	Male	2.5	Positive	Negative	N/A	N/A
Belchertown 1	MAWTD24_167	Ware	Female	3.5+	Positive	Negative	N/A	N/A
Belchertown 1	MAWTD24_168	Pelham	Male	1.5	Positive	Negative	N/A	N/A
Belchertown 1	MAWTD24_169	Ware	Male	3.5+	Positive	Positive	*Babesia odocoile*	PZ528324
Belchertown 1	MAWTD24_170	Williamsburg	Male	2.5	Positive	Negative	N/A	N/A
Belchertown 2	MAWTD24_171	Amherst	Female	2.5	Positive	Negative	N/A	N/A
Belchertown 2	MAWTD24_172	Pelham	Male	3.5+	Positive	Negative	N/A	N/A
Belchertown 2	MAWTD24_173	Belchertown	Male	1.5	Positive	Positive	*Babesia odocoile*	PZ530593
Belchertown 2	MAWTD24_174	Ludlow	Male	2.5	Positive	Positive	*Babesia odocoile*	PZ528325
Belchertown 2	MAWTD24_175	Ludlow	Male	1.5	Positive	Negative	N/A	N/A
Belchertown 2	MAWTD24_176	Belchertown	Male	3.5+	Positive	Negative	N/A	N/A
Belchertown 2	MAWTD24_177	Ware	Male	1.5	Positive	Positive	*Babesia odocoile*	PZ530598
Belchertown 2	MAWTD24_178	Belchertown	Male	0.5	Positive	Positive	*Babesia odocoile*	PZ528346
Belchertown 2	MAWTD24_179	Ware	Male	2.5	Positive	Negative	N/A	N/A
Belchertown 2	MAWTD24_180	Ware	Female	3.5+	Positive	Negative	N/A	N/A
Belchertown 2	MAWTD24_181	Belchertown	Male	1.5	Positive	Negative	N/A	N/A
Belchertown 2	MAWTD24_182	New Salem	Male	2.5	Positive	Positive	*Babesia odocoile*	PZ528397
Belchertown 2	MAWTD24_183	Leverett	Male	2.5	Positive	Negative	N/A	N/A
Belchertown 2	MAWTD24_184	Brimfield	Female	3.5+	Positive	Negative	N/A	N/A
Belchertown 2	MAWTD24_185	Belchertown	Female	0.5	Positive	Positive	*Babesia odocoile*	PZ530597
Belchertown 2	MAWTD24_186	Belchertown	Male	0.5	Positive	Positive	*Babesia odocoile*	PZ530596
Belchertown 2	MAWTD24_187	Granby	Male	1.5	Positive	Negative	N/A	N/A
Belchertown 2	MAWTD24_188	Pelham	Female	2.5	Positive	Negative	N/A	N/A
Belchertown 2	MAWTD24_189	Belchertown	Male	2.5	Positive	Positive	*Babesia odocoile*	PZ530599
Belchertown 2	MAWTD24_190	Granby	Female	3.5+	Positive	Negative	N/A	N/A
Belchertown 2	MAWTD24_191	Granby	Male	0.5	Positive	Positive	*Babesia odocoile*	PZ530600
Ayer	MAWTD24_192	Billerica	Female	0.5	Positive	Negative	N/A	N/A
Ayer	MAWTD24_193	Groton	Male	1.5	Positive	Negative	N/A	N/A
Ayer	MAWTD24_194	Shirley	Female	3.5+	Positive	Negative	N/A	N/A
Ayer	MAWTD24_195	Dracut	Male	1.5	Positive	Positive	*Babesia odocoile*	PZ530601
Ayer	MAWTD24_196	Dracut	Male	3.5+	Positive	Negative	N/A	N/A
Ayer	MAWTD24_197	Dunstable	Male	2.5	Positive	Negative	N/A	N/A

Ayer	MAWTD24_198	Littleton	Female	2.5	Positive	Negative	N/A	N/A
Ayer	MAWTD24_199	Sterling	Male	0.5	Positive	Negative	N/A	N/A
Ayer	MAWTD24_200	Harvard	Female	2.5	Positive	Negative	N/A	N/A
Ayer	MAWTD24_201	Ayer	Male	2.5	Positive	Negative	N/A	N/A
Ayer	MAWTD24_202	Ayer	Male	0.5	Positive	Negative	N/A	N/A
Ayer	MAWTD24_203	Boxborough	Female	1.5	Negative	N/A	N/A	N/A
Ayer	MAWTD24_204	Groton	Female	3.5+	Positive	Negative	N/A	N/A
Ayer	MAWTD24_205	Sudbury	Male	0.5	Positive	Positive	*Babesia odocoile*	PZ528453
Ayer	MAWTD24_206	Sudbury	Female	2.5	Positive	Negative	N/A	N/A
Ayer	MAWTD24_207	Townsend	Male	0.5	Positive	Negative	N/A	N/A
Ayer	MAWTD24_208	Shirley	Female	3.5+	Positive	Negative	N/A	N/A
Ayer	MAWTD24_209	Groton	Female	1.5	Positive	Negative	N/A	N/A
Ayer	MAWTD24_210	Bolton	Male	0.5	Positive	Negative	N/A	N/A
Ayer	MAWTD24_211	Leominster	Male	1.5	Positive	Negative	N/A	N/A
Ayer	MAWTD24_212	Westford	Female	2.5	Positive	Negative	N/A	N/A
Ayer	MAWTD24_213	Stow	Female	1.5	Positive	Negative	N/A	N/A
Ayer	MAWTD24_214	Littleton	Female	1.5	Positive	Negative	N/A	N/A
Ayer	MAWTD24_215	Littleton	Female	1.5	Positive	Negative	N/A	N/A
Ayer	MAWTD24_216	Littleton	Female	1.5	Positive	Negative	N/A	N/A
Ayer	MAWTD24_217	Templeton	Male	1.5	Positive	Negative	N/A	N/A
Ayer	MAWTD24_218	Townsend	Female	3.5+	Positive	Negative	N/A	N/A
Webster	MAWTD24_219	Millbury	Female	0.5	Positive	Negative	N/A	N/A
Webster	MAWTD24_220	Millbury	Male	2.5	Positive	Positive	*Babesia odocoile*	PZ528929
Webster	MAWTD24_221	Uxbridge	Male	0.5	Positive	Negative	N/A	N/A
Webster	MAWTD24_222	Blackstone	Male	1.5	Positive	Negative	N/A	N/A
Webster	MAWTD24_223	Douglas	Male	3.5+	Positive	Negative	N/A	N/A
Webster	MAWTD24_224	Uxbridge	Male	1.5	Positive	Positive	*Babesia odocoile*	PZ528936
Webster	MAWTD24_225	Uxbridge	Male	3.5+	Positive	Negative	N/A	N/A
Webster	MAWTD24_226	Uxbridge	Male	3.5+	Positive	Negative	N/A	N/A
Webster	MAWTD24_227	Uxbridge	Male	0.5	Positive	Positive	*Babesia odocoile*	PZ530602
Webster	MAWTD24_228	Oxford	Male	2.5	Positive	Negative	N/A	N/A
Webster	MAWTD24_229	Charlton	Male	2.5	Positive	Negative	N/A	N/A
Webster	MAWTD24_230	Charlton	Male	1.5	Positive	Negative	N/A	N/A
Webster	MAWTD24_231	Dudley	Male	2.5	Positive	Negative	N/A	N/A
Webster	MAWTD24_232	Dudley	Male	1.5	Positive	Negative	N/A	N/A
Webster	MAWTD24_233	Dudley	Female	2.5	Positive	Negative	N/A	N/A
Webster	MAWTD24_234	Douglas	Male	2.5	Positive	Negative	N/A	N/A
Webster	MAWTD24_235	Dudley	Female	2.5	Positive	Negative	N/A	N/A
Webster	MAWTD24_236	Dudley	Male	3.5+	Positive	Negative	N/A	N/A
Middleton	MAWTD24_237	Wenham	Female	0.5	Positive	Positive	*Babesia odocoile*	PZ528959
Middleton	MAWTD24_238	Hamilton	Male	3.5+	Positive	Negative	N/A	N/A
Middleton	MAWTD24_239	Topsfield	Male	3.5+	Positive	Negative	N/A	N/A
Middleton	MAWTD24_240	Methuen	Female	3.5+	Positive	Negative	N/A	N/A
Middleton	MAWTD24_241	Methuen	Female	1.5	Positive	Negative	N/A	N/A
Middleton	MAWTD24_242	Ipswich	Male	0.5	Positive	Positive	*Babesia odocoile*	PZ529008
Middleton	MAWTD24_243	Essex	Male	2.5	Positive	Negative	N/A	N/A
Middleton	MAWTD24_244	Wenham	Male	1.5	Positive	Negative	N/A	N/A
Hopkinton	MAWTD24_245	Concord	Male	3.5+	Positive	Negative	N/A	N/A
Hopkinton	MAWTD24_246	Franklin	Female	2.5	Negative	N/A	N/A	N/A
Hopkinton	MAWTD24_247	Millbury	Female	2.5	Positive	Negative	N/A	N/A
Hopkinton	MAWTD24_248	Hopkinton	Male	N/A	Positive	Negative	N/A	N/A
Hopkinton	MAWTD24_249	Franklin	Male	0.5	Positive	Negative	N/A	N/A
Hopkinton	MAWTD24_250	Norton	Female	1.5	Positive	Negative	N/A	N/A
Hopkinton	MAWTD24_251	Norton	Male	N/A	Positive	Negative	N/A	N/A
Hopkinton	MAWTD24_252	Halifax	Female	N/A	Positive	Negative	N/A	N/A
Hopkinton	MAWTD24_253	Bellingham	Female	2.5	Positive	Negative	N/A	N/A
Hopkinton	MAWTD24_254	Norfolk	Male	2.5	Positive	Negative	N/A	N/A
Hopkinton	MAWTD24_255	Sherborn	Female	2.5	Positive	Negative	N/A	N/A
Hopkinton	MAWTD24_256	Hopkinton	Male	2.5	Positive	Negative	N/A	N/A
Hopkinton	MAWTD24_257	Mendon	Male	1.5	Positive	Negative	N/A	N/A
Hopkinton	MAWTD24_258	Barre	Male	1.5	Positive	Negative	N/A	N/A
Hopkinton	MAWTD24_259	Wrentham	Female	1	Positive	Positive	*Babesia odocoile*	PZ530741
Hopkinton	MAWTD24_260	Medway	Female	1.5	Positive	Negative	N/A	N/A
Hopkinton	MAWTD24_261	Millis	Male	3.5+	Positive	Negative	N/A	N/A
Hopkinton	MAWTD24_262	Hopkinton	Male	3.5+	Negative	N/A	N/A	N/A
Hopkinton	MAWTD24_263	Norton	Male	2.5	Positive	Negative	N/A	N/A
Hopkinton	MAWTD24_264	Norfolk	Female	3.5+	Positive	Negative	N/A	N/A
Hopkinton	MAWTD24_265	Norton	Male	2.5	Positive	Negative	N/A	N/A

Hopkinton	MAWTD24_266	Westborough	Male	2.5	Positive	Positive	*Sarcocystis* sp.	PZ623933
Hopkinton	MAWTD24_267	Natick	Female	3.5+	Positive	Negative	N/A	N/A
Hopkinton	MAWTD24_268	Franklin	Female	2.5	Positive	Positive	*Babesia odocoile*	PZ533727
Hopkinton	MAWTD24_269	Mendon	Male	1.5	Positive	Negative	N/A	N/A
Hopkinton	MAWTD24_270	Sudbury	Female	2	Positive	Negative	N/A	N/A
Hopkinton	MAWTD24_271	Norton	Female	1.5	Positive	Negative	N/A	N/A
Hopkinton	MAWTD24_272	Norton	Male	1.5	Positive	Negative	N/A	N/A
Hopkinton	MAWTD24_273	Mendon	Male	0.5	Positive	Positive	*Babesia odocoile*	PZ530767
Middleborough	MAWTD24_274	Middleborough	Male	0.5	Positive	Positive	*Babesia odocoile*	PZ530853
Middleborough	MAWTD24_275	Dighton	Male	3.5+	Positive	Negative	N/A	N/A
Middleborough	MAWTD24_276	Middleborough	Male	1.5	Negative	N/A	N/A	N/A
Middleborough	MAWTD24_277	Middleborough	Male	1.5	Negative	N/A	N/A	N/A
Middleborough	MAWTD24_278	Middleborough	Male	1.5	Positive	Positive	*Babesia odocoile*	PZ533726
Middleborough	MAWTD24_279	Middleborough	Male	1.5	Positive	Negative	N/A	N/A
Middleborough	MAWTD24_280	Carver	Female	3.5+	Positive	Negative	N/A	N/A
Middleborough	MAWTD24_281	Wareham	Male	1.5	Positive	Negative	N/A	N/A
Middleborough	MAWTD24_282	Middleborough	Female	3.5+	Positive	Negative	N/A	N/A
Middleborough	MAWTD24_283	Upton	Male	1.5	Positive	Negative	N/A	N/A
Middleborough	MAWTD24_284	Rochester	Male	1.5	Positive	Negative	N/A	N/A
Middleborough	MAWTD24_285	Easton	Female	1.5	Positive	Negative	N/A	N/A
Middleborough	MAWTD24_286	Marshfield	Female	2.5	Positive	Negative	N/A	N/A
Middleborough	MAWTD24_287	Middleborough	Female	3.5+	Positive	Negative	N/A	N/A
Middleborough	MAWTD24_288	Carver	Male	0.5	Positive	Positive	*Sarcocystis* sp.	PZ623934
Middleborough	MAWTD24_289	Carver	Male	1.5	Positive	Negative	N/A	N/A
Middleborough	MAWTD24_290	Pembroke	Male	1.5	Positive	Negative	N/A	N/A
Middleborough	MAWTD24_291	Middleborough	Female	3.5+	Positive	Negative	N/A	N/A
Middleborough	MAWTD24_292	Mansfield	Male	0.5	Negative	N/A	N/A	N/A
Middleborough	MAWTD24_293	Mansfield	Male	3.5+	Positive	Negative	N/A	N/A
Middleborough	MAWTD24_294	Middleborough	Female	3.5+	Positive	Negative	N/A	N/A
Middleborough	MAWTD24_295	Dartmouth	Female	1.5	Positive	Negative	N/A	N/A
Middleborough	MAWTD24_296	Rehoboth	Male	1.5	Positive	Negative	N/A	N/A
Middleborough	MAWTD24_297	East Bridgewater	Female	3.5+	Positive	Negative	N/A	N/A
